# Attitudes towards psychopharmacology and psychotherapy in psychiatric patients with and without migration background

**DOI:** 10.1186/s12888-020-02585-1

**Published:** 2020-04-17

**Authors:** Eva J. Brandl, Nora Dietrich, Nicoleta Mell, Johanna G. Winkler, Stefan Gutwinski, H. Joachim Bretz, Meryam Schouler-Ocak

**Affiliations:** 1 Charité Universitätsmedizin Berlin, corporate member of Freie Universität Berlin, Humboldt-Universität zu Berlin, and Berlin Institute of Health, Department of Psychiatry and Psychotherapy, Campus Mitte, Berlin, Germany Charité Universitätsmedizin Berlin, Berlin, Germany; 2 Psychiatrische Universitätsklinik der Charité im St. Hedwig Krankenhaus, Große Hamburger Str. 5-11, 10115 Berlin, Germany

**Keywords:** Migrants, Attitude, Medication, Psychotherapy

## Abstract

**Background:**

Sociodemographic factors, attitude towards treatment and acculturation may be important factors influencing the decision of immigrants to seek and maintain psychiatric treatment. A better understanding of these factors may significantly improve treatment adherence and outcome in these patients. Therefore, we investigated factors associated the attitude towards psychotherapy and medication in a sample of psychiatric outpatients with and without migration background.

**Methods:**

*N* = 381 patients in a psychiatric outpatient unit offering specialized treatment for migrants were included in this study. Attitude towards psychotherapy was assessed using the Questionnaire on Attitudes Toward Psychotherapeutic Treatment, attitude towards medication with the Drug Attitude Inventory-10. Acculturation, symptom load and sociodemographic variables were assessed in a general questionnaire. Statistical analyses included analyses of covariance and hierarchical regression.

**Results:**

Patients of Turkish and Eastern European origin reported a significantly more positive attitude towards medication than patients without migration background. When controlling for sociodemographic and clinical variables, we did not observe any significant differences in attitude towards psychotherapy. Acculturation neither influenced the attitude towards psychotherapy nor towards medication.

**Conclusion:**

Our study indicates that sociodemographic and clinical factors may be more relevant for patients´ attitudes towards treatment than acculturation. Considering these factors in psychiatric treatment of patients with migration background may improve treatment outcome and adherence.

## Background

With rising numbers of migrants and refugees over the past years, there has been increasing interest in mental health issues of these groups. A variety of psychosocial risk factors, including lower socioeconomic status, higher risk for unemployment [1], discrimination [2] and experience of violence as well as migration stress [3] contribute to higher rates of psychiatric disorders in migrant populations. Although the risk for specific psychiatric disorders varies depending on the region of origin [3] as well as on the circumstances of being a migrant or a refugee [4], generally a higher prevalence of most psychiatric disorders has been reported [5–12]. Despite this increased risk and a higher symptom load compared to individuals without migration background [13–15], migrants tend to use mental health services, including psychotherapy, less often [16–19]. In addition, treatment adherence to psychopharmacological treatment has been reported to be lower in migrants and ethnic minorities [20–24]. Insufficient consideration of sociodemographic differences between migrants and non-migrants seeking treatment [15, 25] in clinical practice as well as relevant language and cultural barriers provide partial explanations for these issues. Another important, yet insufficiently investigated factor influencing treatment seeking and adherence is the attitude towards psychiatric and psychotherapeutic treatment in migrants. A negative attitude towards psychotherapy may be one of the main reasons not to seek treatment [26]. Only few studies on attitude towards psychotherapy in migrants have been performed to date, and most of these have been conducted in the United States, indicating a generally less positive attitude towards psychotherapy in migrants. A high impact of sociodemographic and symptom-related factors on the attitude has been reported [27]. Acculturation of migrants has also been identified as a factor influencing attitude towards psychotherapy [28–30]. However, a recent meta-analysis found ethnic differences in the impact of acculturation on attitudes towards psychological treatment with little impact in most ethnic groups except for individuals of Asian heritage [31]. Literature on the attitude towards psychotherapy in migrant populations in Germany and Europe is sparse but also indicates a less positive attitude in these groups [32–35]. However, the influence of acculturation on attitude towards psychotherapy of migrants in Germany has not been investigated extensively yet. Education, age, sex, (e.g., [27, 36, 37]) as well as psychiatric symptom load (e.g., [36, 38, 39]) have been investigated regarding an influence on attitude towards psychotherapy with heterogeneous results, indicating a need for further studies in this field.

Attitude towards medication has been shown to be an important predictor of medication adherence, e.g. [40–42]. The attitude towards pharmacological treatment in migrants and ethnic minorities has only been examined in a few studies. Similar to the attitude towards psychotherapy, a less positive attitude towards medication has been found in ethnic minority patients [43–48].

The influence of acculturation on medication adherence in patients with mental disorders has not been investigated extensively, but better adherence in individuals with stronger orientation towards the host culture has been reported [49, 50]. However, acculturation was not associated with attitude towards medication in all studies [51]. In other medical areas, acculturation has been associated with better drug adherence, e.g. [49, 52–54]. To the best of our knowledge, there are no data on the influence of acculturation of attitude towards medication in psychiatric patients with migration background in Germany.

In summary, attitude towards psychotherapy and medication may influence treatment adherence and outcome. However, the specific relevance of factors potentially influencing these attitudes towards treatment, including clinical and sociodemographic factors, migration background and acculturation in migrants is not well understood yet. Therefore, we set out to a) examine the attitude of psychiatric patients with and without migration background towards psychotherapy as well as towards medication and b) to identify the association of relevant sociodemographic and clinical factors and acculturation with the attitudes towards psychotherapy and medication.

## Methods

### Participants

All patients treated in the outpatient unit of the Psychiatric University Hospital of Charité at St.Hedwig-Hospital in Berlin, Germany, between April and June 2015 and who did not fulfill our exclusion criteria (acute psychosis, severe cognitive impairment, acute emergency treatment) were invited to fill out a questionnaire provided in seven languages (German, English, French, Arabic, Farsi, Turkish and Russian). The outpatient unit offers general psychiatric outpatient treatment to two large downtown districts of Berlin and additionally is specialized in treatment of patients with migration background.

Questionnaires were handed out to the patients who came to their appointments in the outpatient unit and filled out in the waiting area after informed consent was obtained. Information on current medication and diagnoses according to ICD-10 criteria was obtained from electronic medical records. The study was approved by the ethics board of Charité - Universitätsmedizin Berlin and conducted in accordance with the Declaration of Helsinki. All participants gave written informed consent before participation in the study.

### Measures

The questionnaire contained a general part with demographic and clinical data (such as marital status, duration of illness, employment status etc.). Current symptom load was assessed with the Symptom Checklist 14 (SCL-14), a short version of the Symptom Checklist 90 [55]. These general characteristics of the sample have been described previously [15]. The SCL-14 subscales reached Cronbach’s alpha of α = 0.89 for somatization, α = 0.83 for anxiety and α = 0.87 for depression in our dataset. For the purpose of this study, only patients without migration background and the largest migrant groups (Turkish, Eastern European, middle Eastern/north African (MENA [56];) plus Afghanistan/Pakistan (MENAP)) migration background) were included since the other groups were too small for meaningful analyses.

#### Attitude towards psychotherapy

Attitude towards psychotherapy was assessed using the Questionnaire on Attitudes Toward Psychotherapeutic Treatment (QAPT [36]) which consists of 20 statements rated on a Likert-type scale ranging from 1 (“I do not agree”) to 4 (“I agree”). Four subscales are created to assess the attitude towards psychotherapy: psychotherapist’s *competence*, anticipated *judgment* by others, *general attitude* towards psychotherapy and personal *acceptance*. Higher scores indicate a more positive attitude toward psychotherapy. The validity of the instrument was confirmed in the original publication of the questionnaire. The internal consistency of the subscales has been confirmed in the original publication [36]. In our own data set, the QAPT subscales reached the following *α-*values: competence: α = 0.52, judgment: α = 0.64, general attitude: α = 0.58, acceptance: α = 0.61. The QAPT has been used in other cross-cultural studies on attitude towards psychotherapy before with higher α-values for the QAPT subscales in some studies [35, 57] and comparable α-values to our sample in others [34].

#### Attitude towards medication

To examine attitudes towards and subjective experience with medication, we applied the 10-item version of the Drug Attitude Inventory (DAI [58]). The scale consists of ten statements (for example: “For me, the good things about medication outweigh the bad”; “I feel more normal on medication”; “It is ununatural for my mind and body to be controlled by medication”) with a dichotomous response option (true/ false) and assesses general attitude towards medication. Several studies have underlined the validity and reliability of the DAI [59]. Cronbach’s *α* of the DAI in our dataset was 0.68.

#### Acculturation

In patients with migration background (defined as not holding German citizenship per birth, having immigrated to Germany and/or having at least one parent not holding German citizenship following the definition of the Federal Statistical Office [1]), acculturation was assessed using the Acculturation Index by Ward & Rana-Deuba [60]. Based on a two-dimensional approach to acculturation it contains two subscales: “*host national identification”* and “*co-national identification”*. Both scales range between 1 and 7 with higher values indicating a stronger identification with that culture. A high reliability *(co-national identification scale* α = .93 and *host identification scale* α = .96) and good validity of the Acculturation Index has previously been reported [61] with the same α-values being obtained in our own dataset .

### Statistical analyses

Data were analyzed using *RStudio 0.99.489* for *Windows*. Differences between the included migrant groups and patients without migration background in sociodemographic and clinical parameters were explored with analysis of variance (ANOVA), Chi-Quadrat-tests and Fisher-Yates-tests, respectively.

Analyses of covariance (ANCOVA) were conducted in order to assess if the subsamples with migration background differed on the five dimensions (four QAPT scales and DAI) from the subsample without migration background. Potentially relevant covariates (SCL-14 subscale values for anxiety, somatization and depression; age; education; gender; religious affiliation; medication intake; psychiatric inpatient stays) were theoretically derived, e.g. [27, 30]. Only those covariates that showed a significant correlation with the respective dependent variable (QAPT subscales and DAI) were included in the final analyses and are provided for each analysis in Table 2. Two ANCOVA were conducted per dimension. Due to the gender distribution differences in our subsamples, the first analysis included only gender as covariate in case it correlated with the dependent variable. The second analysis also included further sociodemographic (e.g. education, religious affiliation) and clinical factors (e.g. symptom severity, inpatient stays, medication intake). The adjusted means were compared with the Dunnett-test using the sample without migration background as control.

Hierarchical regressions were conducted to test if acculturation predicts a significant additional amount of variance in the samples with migration background after accounting for sociodemographic and clinical variables. The covariates from the prior analysis were adopted for each dependent variable. In the second step both scales of the Acculturation Index were added. Due to the exploratory character of the analyses, we did not correct *p*-values for multiple testing. Patients who had returned questionnaires with more than 20% of missing values were excluded from the analyses. In the total sample, 6.6% of values were missing. We applied listwise deletion to missing values for the ANCOVA and the hierarchical regression to avoid a high loss of information.

## Results

### Sociodemographic data

The original sample comprised *N* = 423 participants who had returned completed questionnaires out of *N* = 700 patients who were invited to participate in the study response rate of 60.5% [15]. Due to the limited sample sizes, patients from Asia (*N* = 5), Africa (*N* = 10) Western Europe and America (*N* = 19) were not included in the analyses. *N* = 8 patients had to be excluded due to incomplete questionnaires, resulting in a total sample of *N* = 381 individuals. The sample included patients without migration background (*N* = 194), and patients of Turkish (*N* = 111), Eastern European (*N* = 39) or MENAP (*N* = 37) background. We found significant differences in terms of gender, education, religiousness, medication intake and diagnoses among the subsamples (see Table 1) as previously described for the overall sample [15]. There were also significant differences in reported symptom severity regarding somatic and anxiety symptoms. Due to the observed differences, sociodemographic and clinical variables were incorporated in the following statistical analyses as covariates.

**Table 1:** Sociodemographic and symptom related characteristics of the total sample and the four subsamples

Variable	Total sample *n* = 381 *n* (%) /M ± SD	Sample without migration background *n* = 194 *n* (%) /M ± SD	Sample with Turkish migration background *n* = 111 *n* (%) /M ± SD	Sample with East European migration background *n* = 39 *n* (%) /M ± SD	Sample with MENAP migration background *n* = 37 *n* (%) /M ± SD	*p*-value
Gender						
female	224 (58.8)	100 (51.6)	85 (76.6)	21 (53.9)	18 (48.6)	< .001*
male	157 (41.2)	94 (48.5)	26 (23.4)	18 (46.2)	19 (51.4)	
Age	43.9 (12.5)	44.3 (13.4)	45.3 (10.9)	43.2 (13.2)	38.2 (10.5)	.061
School education						.009*
high	217 (57.0)	126 (66.0)	48 (43.2)	25 (64.1)	18 (48.6)	
low	138 (36.2)	60 (30.9)	52 (46.8)	12 (30.8)	14 (37.8)	
not indicated	26 (6.8)	8 (4.1)	11 (9.9)	2 (5.1)	5 (13.5)	
Country of birth						
Germany	223 (58.5)	193 (99.5)	19 (17.1)	7 (17.9)	4 (10.8)	
Other country	158 (41.5)	1 (USA)	92 (Turkey)	30 (82.1) (3 Bulgaria, 1 Czech Republic, 1 Hungary, 1 Kosovo, 1 Latvia, 10 Poland, 1 Rumania, 8 Russia, 4 Serbia, 1 Ukraine, 1 former Yugoslavia)	33 (89.2) (4 Iraq, 2 Iran, 1 Israel, 6 Lebanon, 2 Morocco, 1 Pakistan, 16 Syria, 1 Tunisia)	
Religious affiliation						< .001*
Yes	230 (60.4)	76 (39.2)	97 (87.4)	25 (64.1)		
No	122 (32.0)	94 (48.5)	11 (9.9)	13 (33.3)		
Not indicated	29 (7.6)	24 (12.4)	3 (2.7)	1 (2.6)		
Medication intake						.597
Yes	328 (86.1)	160 (82.5)	98 (88.3)	35 (89.7)	35 (34.6)	
No	53(13.9)	34 (17.5)	13 (11.7)	4 (10.3)	2 (5.4)	
Number of medications	1.5 (0.7)	1.3 (1.0)	1.5 (0.9)	1.7 (1.1)	1.5(0.7)	.014*
Type of medication						.003*
Antidepressant	269(70.6)	109 (56.2)	98 (88.3)	31 (79.5)	31 (83.8)	
Neuroleptic	207 (54.3)	111 (57.2)	50 (45.0)	22 (56.4)	24 (64.9)	
Tranquillizer	23 (6.9)	13 (6.7)	5 (4.5)	4 (10.3)	1 (2.7)	
Mood stabilizer	37 (7.1)	20 (10.3)	8 (7.2)	9 (23.1)	0 (0.0)	
Antidementiva	0(0.0)	0 (0.0)	0 (0.0)	0 (0.0)	0 (0.0)	
Pain Medication	12 (4.3)	2 (1.0)	8 (7.2)	2 (5.1)	0 (0.0)	
Internal medication	56 (14.7)	35 (18.0)	15 (13.5)	3 (7.7)	3 (8.1)	
Substitution/ addiction treatment medication	1 (0.3)	1 (0.5)	0 (0.0)	0 (0.0)	0 (0.0)	
Other	12 (3.1)	5 (2.6)	5 (4.5)	0 (0.0)	2 (5.4)	
Inpatient stays (psychiatry)						.005*
never	132 (34.6)	49 (25.3)	55 (49.5)	7 (17.9)	21 (56.7)	
seldom	144 (37.8)	76 (39.2)	38 (34.2)	20 (51.3)	10 (27.0)	
often	92 (24.1)	63 (31.5)	14 (12.6)	10 (25.6)	5 (13.5)	
not indicated	13 (3.4)	6 (3.1)	4 (3.6)	2 (5.1)	1 (2.7)	
Symptom severity						
SCL Somatization	2.4 (1.2)	2.0 (1.0)	3.1 (1.3)	2.4 (1.2)	2.6 (1.0)	< .001*
SCL Depression	2.8(1.1)	2.6 (1.0)	3.0 (1.1)	3.2 (1.2)	3.0 (1.0)	.184
SCL Anxiety	2.0 (1.0)	1.7 (0.9)	2.3 (1.1)	1.9 (1.1)	2.5 (1.2)	< .001*
Diagnosis (ICD-10)						
F0	8 (2.1)	7 (3.6)	1 (0.9)	0 (0.0)	0 (0.0)	< .001*
F1	85 (30.2)	65 (33.5)	9 (8.1)	5 (12.8)	6 (16.2)	
F2	86 (30.6)	51 (26.3)	18 (16.2)	9 (23.1)	8 (21.6)	
F3	164 (43.0)	79 (40.7)	49 (44.1)	15 (38.5)	21 (56.8)	
F4	141 (37.0)	50 (25.8)	61 (55.0)	15 (38.5)	15 (40.5)	
F5	7 (1.8)	3 (1.5)	2 (1.8)	2 (5.1)	0 (0.0)	
F6	61 (16.0)	46 (23.7)	7 (6.3)	8 (20.5)	0 (0.0)	
F7	7 (1.8)	7 (3.6)	0 (0.0)	0 (0.0)	0 (0.0)	
F8	0(0.0)	0 (0.0)	0 (0.0)	0 (0.0)	0 (0.0)	
F9	2 (0.5	1 (0.5)	1 (0.9)	0 (0.0)	0 (0.0)	

*Note.* Low level of school education was defined as 0–9 years of school, a high level of school education as 10–13 years of school education (following Petrowski und Kollegen, 2014). Regarding the classification of inpatient stays, 1–2 inpatient stays were catigorized as seldom and more than 2 as often. MENA = Middle East and North Africa region, SCL = *Symptom-Check-List*, *n* = sample size, *M =* arithmetic mean, *SD* = standard deviation. *Note.* The number of diagnosis does not correspond with the sample size as some patients have multiple diagnosis. The diagnosis have been classified according to the *International Classification of Diseases 10* (Graubner, 2014). F0 = Organic, including symptomatic, mental disorders, F1 = Mental and behavioural disorders due to use of psychoactive substances, F2 = Schizophrenia, schizotypal and delusional disorders, F3 = Mood/ affective disorders, F4 = Neurotic, stress-related and somatoform disorders, F5 = Behavioural syndromes associated with physiological disturbances and physical factors, F6 = Disorders of personality and behaviour in adult persons, F7 = Mental retardation, F8 = Disorders of psychological development, F9 = Behavioural and emotional disorders with onset usually occurring in childhood and adolescence

* *p* < .05

### Attitudes toward psychotherapy and medication

First, we analyzed whether patients with Turkish, Eastern European and MENAP background differed significantly in their attitude towards psychotherapy as measured by the four scales of the QAPT and in their attitude towards medication measured by the DAI as compared to patients without migration background. Two ANCOVA were conducted per QAPT scale and DAI. In the first ANCOVA, we only controlled for gender if necessary. In the second ANCOVA, we also added further relevant sociodemographic and clinical control variables. Sociodemographic and clinical variables with significant association with at least one of the QAPT subscales were education, number of inpatient stays in the history, current symptom load on the SCL subscales somatization and depression.

The mean value of the QAPT-judgment scale was significantly lower among the samples with East European and MENAP background compared to the sample without migration background, indicating a less positive attitude on this subscale of the QAPT (see Supplementary Table S1). However, after controlling for sociodemographic variables, no significant differences remained. On the QAPT scales competence, acceptance and general attitudes, the samples with Eastern European, Turkish and MENAP background did not differ significantly from the sample without migration background in both analyses (see Supplementary Tables S2-S4).

Regarding the attitude towards medication, patients with Turkish and Eastern European background had a significantly more positive attitude compared to the sample without migration background. This remained significant after controlling for potentially relevant sociodemographic and clinical variables (see Supplementary Table S5). There was no statistically significant difference in attitude towards medication between the MENAP-subgroup and patients without migration background.

### Acculturation and attitudes

In the next step, we examined if acculturation explained an additional amount of variance beyond the identified relevant sociodemographic and clinical variables. We conducted a hierarchical regression with the two scales of the acculturation index (host national identification and co-national identification) added in the second step. The main results are presented in Table 2 (for further details, see Supplementary Table S6). The first *p*-value indicates if the model explains a significant amount of variance as compared to a null model. The second p-value indicates whether the second model including the acculturation index (step 2) explains significantly more variance than the model without the acculturation index (step 1). For reasons of simplicity only the test statistics of the additional variables are presented in the table. The F-tests for ∆R^2^ did not reach significance (with one exception in the East European sample on the QAPT-judgment scale). Hence, the models including the acculturation indexes (apart from one exception) did not explain significantly more variance than the models without the acculturation indexes, indicating no major association of acculturation with the attitude towards psychotherapy as well as towards medication in our sample.

**Table 2:** Association of acculturation with attitude towards psychotherapy and medication

	Turkish background (*N* = 111)					Eastern European background (*N* = 39)					MENAP background (*N* = 37)				
Dependent Variable	*R*^*2*^	*∆R*^*2*^	*F* for *∆R*^*2*^	*p* for *∆R*^*2*^	*n*	*R*^*2*^	*∆R*^*2*^	*F* for *∆R*^*2*^	*p* for *∆R*^*2*^	*n*	*R*^*2*^	*∆R*^*2*^	*F* for *∆R*^*2*^	*p* for *∆R*^*2*^	*n*
**QUAPT judgment**															
**Step 1**:	.17*	.17	3.83	.003*		.27	.27	1.66	.186		.16	.16	0.86	.523	
**Step 2:**	.20*	.03	1.91	.154		.50	.23	4.61	.022*		.19	.03	0.42	.662	
Host national identification															
Co-national identification															
					100					26					29
**QUAPT competence**															
**Step 1:**	.03	.03	1.36	.263		.12	.12	1.50	.244		.07	.07	1.07	.359	
**Step 2:**	.06	.03	1.17	.315		.23	.11	1.54	.237		.07	.00	0.01	.994	
Host national identification															
Co-national identification															
					90					26					30
**QUAPT-acceptance**															
**Step 1:**	.07	.07	3.09	.051		.35*	.35	6.10	.008*		.01	.01	0.11	.897	
**Step 2:**	.12*	.05	2.56	.083		.39*	.04	.76	.480		.04	.03	0.42	.663	
Host national identification															
Co- national Identification															
					90					26					31
**QUAPT general attitude**															
**Step 1**:	.19*	.19	3.12	.008*		.47*	.47	2.71	.047*		.02	.02	0.07	.998	
**Step 2:**	.19*	.00	0.34	.715		.51	.04	0.51	.607		.10	.08	0.81	.459	
Host national identification															
Co-national identification															
					89					25					27
**Drug Attitude Inventory**															
**Step 1:**	.09	.09	2.37	.058		.28	.28	2.24	.096		.19	.19	1.57	.212	
**Step 2:**	.09	.00	0.04	.966		.34	.06	0.87	.432		.20	.02	0.14	.871	
Host national identification															
2003Co-national identification															
					98					28					31

Main results of the hierarchical regression predicting the QUAPT scales judgment, competence, acceptance and general attitude as well as the DAI scale in the samples with Turkish, East European and MENAP background. The association of control variables with attitude towards psychotherapy and medication are included in Step 1. Acculturation scales are added to the other variates in the second step. For simplicity reasons, the control variables as well as the B- and β-values are not shown in this Table, details can be found in Supplementary Table S6. MENA = Middle East and North Africa Region, QUAPT = *Questionnaire on Attitudes Toward Psychotherapeutic Treatment*, DAI = *Drug Attitude Inventory*

* *p* < .05

## Discussion

To the best of our knowledge, this is the first study to investigate attitude towards psychotherapy and medication in a sample of patients with and without migration background in a psychiatric outpatient unit. We did not find major differences in the attitude towards psychotherapy after controlling for relevant sociodemographic and clinical factors. The attitude towards medication was more positive in patients with Turkish and Eastern European background. Acculturation did not have a significant association with patients´ attitudes towards treatment in our sample except for the QAPT-judgment scale in the Eastern European subsample. In this subsample, a higher level of acculturation was associated with a more positive attitude towards psychotherapy regarding anticipated judgment by others. However, due to the very limited sample size of this subsample and the low Cronbach’s *α* of the QAPT-judgment scale, this finding needs to be considered with caution. Since this association was not observed in the two other subsamples with MENAP- and Turkish background, we do not assume a major impact of acculturation on anticipated judgement for utilizing psychotherapy by others; however, a replication in a larger sample would be required before final conclusions can be drawn.

These findings are partially in line with results of previous studies. Calliess et al. [32] also did not report an impact of acculturation on the attitude towards psychotherapy in young adult individuals with Turkish background in Germany. However, they found a significant influence of migration background on the attitude towards psychotherapy after controlling for sociodemographic variables, whereas these differences did not remain significant after controlling for confounders in our sample. In most ethnic groups, a recent meta-analysis did not report a major impact of acculturation as well [31]. Knipscheer & Kleber [33] reported significant differences between migrants and non-migrants in their attitude towards psychotherapy in a Dutch sample; however, while statistically significant, the observed differences were rather small. Ditte et al. reported a less favorable attitude towards psychotherapy in Russian migrants as compared to German participants [35]. Our group found a less positive attitude towards psychotherapy in individuals of Turkish background in a previous study [34], where migration background was the most important predictor beyond sociodemographic factors. Nonetheless, the participants in the previous study were recruited in waiting rooms of general practitioners whereas the participants for the current study were already in psychiatric treatment, which may in parts explain the observed differences in the results. It can be hypothesized that patients already actively seeking psychiatric treatment in general may have a more positive attitude towards psychiatry and psychotherapy than individuals not seeking psychiatric treatment and that therefore migration background may play a smaller role in our sample than in samples from the general population. In addition, the outpatient unit from which patients were recruited for the study is specialized in treatment of migrants. The use of professional interpreters and the presence of staff with migration background may reduce feelings of stigmatization and could also contribute to a less negative view on psychotherapy in patients with migration background.

The finding that sociodemographic and clinical variables influence attitude towards psychotherapy is in line with previous studies. For example, Constantine and Gainor [39] found that individuals with higher depression symptom load were more likely to seek treatment. When correcting for education level, differences in attitude towards medication were smaller. Attitude towards treatment is generally considered to be more positive in patients with higher education levels (e.g., [27, 36]). Gender only partially predicted attitude towards psychotherapy in our analyses, which is in line with mixed findings of previous studies [33, 37, 62].

The attitude towards medication was more positive in patients of Turkish and Eastern European background. While gender, depression symptom load and current medication intake were associated with attitude towards medication in our sample, acculturation was, similar to the attitude towards psychotherapy, not a significant predictor. The more positive attitude in these two subgroups contradicts other studies which reported a less favorable attitude towards medication in ethnic minorities [43–48]*.* However, most of the previous studies have been conducted in the US examining individuals of Hispanic or African-American origin. One study conducted in Switzerland included mainly immigrants from Western European countries who were excluded from our analyses due to the small sample size in our sample [48]. Therefore, our result indicates cultural differences in attitude towards medication and underlines the importance in considering specific cultural factors when initiating medication in psychiatric patients with migration background. The finding that acculturation did not influence attitude towards medication beyond sociodemographic factors is in line with an earlier study in Hispanic patients [51]. However, since other studies found an impact of acculturation on medication adherence [49, 50, 52–54]*,* which may in parts represent attitude towards medication, final conclusions cannot be drawn and more research in this field is required.

Several limitations need to be considered in interpretation of our findings. The sample was a convenience sample and not a representative data set, so the results cannot be applied to the general population. In particular, since the participants were all patients in a psychiatric outpatient unit, conclusions about reasons for migrants to not utilize psychiatric treatment cannot be drawn. In addition, the sample size of the subgroups was rather small, limiting statistical power to identify significant effects. Due to the small sample size, duration of stay in Germany and comparisons between 1st vs. 2nd migrant generation could not be incorporated in our analyses. Subgroup analyses by type of medication or psychiatric diagnose could also not be performed due to the limited sample size. Although we controlled for confounding variables in our analyses, the results may be biased due to other differences among the groups. The questions in the DAI were related to general attitude towards medication and not to psychopharmacology specifically; therefore, the attitude towards specific antidepressant or antipsychotic treatment cannot be assessed with our data. Finally, the Cronbach’s alpha of the QAPT subscales and the DAI in our sample was not very high, indicating low reliability and limiting the ability to detect significant differences.

## Conclusions

In summary, our study contributes to a better understanding of views on psychotherapy and medication in migrants. Since sociodemographic differences among different migrant groups and patients without migration background seem to be stronger associated with patients´ views as compared to acculturation, our study underlines the need to consider these sociodemographic factors in psychiatric treatment of migrants.

## Supplementary information

**Additional file 1: **Supplementary Tables. **Tables S1–5** Results and descriptive statistics of the two analysis of covariance with the factor migration background and the Drug Attitude Inventory (DAI) as dependent variable. R^2^ = .19*, corrected R^2^ = .17 (both for analysis 2). The corrections are based on the mean value of SCL Somatization M = 2.41, SCL Depression M = 2.83, SCL Anxiety M = 1.99. The DAI value represents an arithmetic mean of a 2 point Likert scale (1 = True, 2 = False) with higher values indicating a more positive attitude. MENA = Middle East and North Africa Region, MG = migration background, DAI = Drug Attitude Inventory, SCL = Symptom Check List, SE = standard error, Sum Sq = Sum of Squares, df = degrees of freedom, MSS = Mean sum of squares. **Table S6**. Complete results of the hierarchical Regression predicting the QUAPT scales judgment, competence, acceptance and general attitude as well as the DAI scale within the samples with Turkish, East European and MENAP background. The acculturation scales are added in the second step. School education: 0 = low, 1 = high, Gender: 0 = female, 1 = male, religious affiliation: 0 = yes, 1 = no. Higher scores on the scales of the QUAPT and DAI indicate a more positive attitude on that scale. For simplicity reasons the control variables are only presented in step 1. MENA = Middle East and North Africa Region, QUAPT = Questionnaire on Attitudes Toward Psychotherapeutic Treatment, DAI = Drug Attitude Inventory, SCL = Symptom Check List.* *p* < .05

## Data Availability

The datasets used for the current study are available from the corresponding author on reasonable request.

## Abbrevations

ANCOVA: Analysis of covariance
DAI: Drug attitude inventory
MENA: Middle East, North Africa
MENAP: Middle East, North Africa, Afghanistan/Pakistan
QAPT: Questionnaire on attitudes toward psychotherapeutic treatment
SCL-14: Symptom Checklist-14
