# Supplementary material for: Attitudes towards psychopharmacology and psychotherapy in psychiatric patients with and without migration background
Source: BMC Psychiatry. 2020 Apr 17;20:176. doi: 10.1186/s12888-020-02585-1 (PMC7164188; doi:10.1186/s12888-020-02585-1)
Supplement: Supplementary file 1 — Additional file 1: Supplementary Tables. Tables S1–5 Results and descriptive statistics of the two analysis of covariance with the factor migration background and the Drug Attitude Inventory (DAI) as dependent variable. R2 = .19*, corrected R2 = .17 (both for analysis 2). The corrections are based on the mean value of SCL Somatization M = 2.41, SCL Depression M = 2.83, SCL Anxiety M = 1.99. The DAI value represents an arithmetic mean of a 2 point Likert scale (1 = True, 2 = False) with higher values indicating a more positive attitude. MENA = Middle East and North Africa Region, MG = migration background, DAI = Drug Attitude Inventory, SCL = Symptom Check List, SE = standard error, Sum Sq = Sum of Squares, df = degrees of freedom, MSS = Mean sum of squares. Table S6. Complete results of the hierarchical Regression predicting the QUAPT scales judgment, competence, acceptance and general attitude as well as the DAI scale within the samples with Turkish, East European and MENAP background. The acculturation scales are added in the second step. School education: 0 = low, 1 = high, Gender: 0 = female, 1 = male, religious affiliation: 0 = yes, 1 = no. Higher scores on the scales of the QUAPT and DAI indicate a more positive attitude on that scale. For simplicity reasons the control variables are only presented in step 1. MENA = Middle East and North Africa Region, QUAPT = Questionnaire on Attitudes Toward Psychotherapeutic Treatment, DAI = Drug Attitude Inventory, SCL = Symptom Check List.* p < .05 [file 12888_2020_2585_MOESM1_ESM.docx]

**Supplementary Tables**

|  | QUAPT Judgment | | | | | | | | |
| --- | --- | --- | --- | --- | --- | --- | --- | --- | --- |
| Migration background | Corrected estimated mean value (corrected SE) | | | | | | *p*-value | | |
|  | Analysis 1 | | | Analysis 2 | | | Analysis 1 | Analysis 2 | |
| No MB | 2.80 (0.13) | | | 2.73 (0.06) | | |  | |  |
| Turkish MB | 2.60 (0.05) | | | 2.67 (0.07) | | | .056 | | .892 |
| Eastern European MB | 2.36* (0.09) | | | 2.46 (0.11) | | | .002* | | .089 |
| MENAP MB | 2.27* (0.13) | | | 2.45 (0.12) | | | < .001* | | .082 |
| Variable | | Sum Sq | *Df* | | MSS | F | *p*-value | | |
| Covariates analysis 1:  ------- | | --- | *----* | | --- | --- | *---* | | |
| Independent variable: | |  |  | |  |  |  | | |
| Migration background | | 12.58 | 3 | | 4.19 | 8.15 | < .001* | | |
| Residuals | | 186.91 | 363 | | 0.51 |  |  | | |
| Covariates analysis 2:  SCL Somatization  SCL Anxiety | | 13.80  0.98 | 1  1 | | 13.80  0.98 | 30.90  2.20 | < .001*  .139 | | |
| SCL Depression | | 8.44 | 1 | | 8.44 | 18.92 | < .001* | | |
| Religious Affiliation  Medication intake  Independent variable: | | 0.43  0.65 | 1  1 | | 0.43  0.65 | 0.97  1.46 | .325  .228 | | |
| Migration background | | 3.67 | 3 | | 1.22 | 2.74 | .044* | | |
| Residuals | | 144.69 | 324 | | 0.45 |  |  | | |

**Table S1.** Results and descriptive statistics of the two analyses of covariance with the factor migration background and the QUAPT judgment scale as dependent variable. *R*² = .16*, corrected *R*² = .14 (both for analysis 2). The corrections are based on the mean value of SCL Somatization *M*=2.41, SCL Depression *M* = 2.83, SCL Anxiety *M* = 1.99. MENAP = Middle East and North Africa Region plus Afghanistan/Pakistan, MB = migration background, QUAPT = *Questionnaire on Attitudes Toward Psychotherapeutic Treatment*, SCL = *Symptom Check List, SE =* standard error, Sum Sq = Sum of Squares, df = degrees of freedom, MSS = Mean sum of squares.

* *p* < .05

|  | QUAPT Competence | | | | | | | | |
| --- | --- | --- | --- | --- | --- | --- | --- | --- | --- |
| Migration background | Corrected estimated mean value (corrected *SE)* | | | *p*-value | | | | | |
|  | Analysis 1 | | Analysis 2 | Analysis 1 | | Analysis 2 | | |  |
| No MB | 3.30 (0.04) | | 3.30 (0.04) |  | |  | | |  |
| Turkish MB | 3.25 (0.05) | | 3.26 (0.05) | .826 | | .927 | | |  |
| Eastern European MB | 3.39 (0.09) | | 3.37 (0.09) | .704 | | .844 | | |  |
| MENAP MB | 3.51 (0.09) | | 3.49 (0.10) | .096 | | .175 | | |  |
| Variable | | Sum Sq | *Df* | | MSS | | F | *p*-value | |
| Covariates analysis 1:  ------- | | --- | *---* | | --- | | --- | *---* | |
| Independent variable:  Migration background | | 1.962 | 3 | | 0.65 | | 2.37 | 0.07 | |
| Residuals | | 99.56 | 361 | | 0.28 | |  |  | |
| Covariates analysis 2: | |  |  | |  | |  |  | |
| School education | | 2.68 | 1 | | 2.68 | | 10.09 | .002* | |
| Inpatient stays  Independent variable: | | 1.19 | 1 | | 1.19 | | 4.49 | .035* | |
| Migration background | | 1.36 | 3 | | 0.45 | | 1.71 | .166 | |
| Residuals | |  |  | |  | |  |  | |

**Table S2.** Results and descriptive statistics of the two analyses of covariance with the factor migration background and the QUAPT competence scale as dependent variable. *R*² = .06*, corrected *R*² = .04 (both for analysis 2). The corrections are based on the mean value of SCL Somatization *M*=2.41, SCL Depression *M* = 2.83, SCL Anxiety *M* = 1.99. MENAP = Middle East and North Africa Region plus Afghanistan/Pakistan, MB = migration background, QUAPT = *Questionnaire on Attitudes Toward Psychotherapeutic Treatment*, SCL = *Symptom Check List, SE =* standard error, Sum Sq = Sum of Squares, df = degrees of freedom, MSS = Mean sum of squares.

* *p* < .05

|  | QUAPT Acceptance | | | | | | | | |
| --- | --- | --- | --- | --- | --- | --- | --- | --- | --- |
| Migration background | Corrected estimated mean value (corrected *SE)* | | | | | *p*-value | | | |
|  | Analysis 1 | | | Analysis 2 | | Analysis 1 | | Analysis 2 | |
| No MB | 3.41 (0.04) | | | 3.41 (0.04) | |  | |  | |
| Turkish MB | 3.44 (0.05) | | | 3.45 (0.05) | | .957 | | .951 | |
| Eastern European MB | 3.42 (0.09) | | | 3.41 (0.09) | | 1.000 | | 1.000 | |
| MENAP MB | 3.52 (0.09) | | | 3.45 (0.09) | | .625 | | .986 | |
| Variable | | Sum Sq | *Df* | | MSS | | F | | *p*-value |
| Covariates analysis 1:  ------------------ | | --- | *---* | | --- | | --- | | *---* |
| Independent variable:  Migration background | | 0.34 | 3 | | 0.11 | | 0.40 | | 0.751 |
| Residuals | | 101.30 | 362 | | 0.28 | |  | |  |
| Covariates analysis 2:  School education | | 4.89 | 1 | | 4.89 | | 18.76 | | < .001* |
| Inpatient stays  Independent variable: | | 2.48 | 1 | | 2.48 | | 9.51 | | .002* |
| Migration background | | 0.07 | 3 | | 0.02 | | 0.09 | | .963 |
| Residuals | | 84.91 | 336 | | 0.26 | |  | |  |

**Table S3.** Results and descriptive statistics of the two analyses of covariance with the factor migration background and the QUAPT acceptance scale as dependent variable. *R*² = .08*, corrected *R*² = .07 (both for analysis 2). The corrections are based on the mean value of SCL Somatization *M*=2.41, SCL Depression *M* = 2.83, SCL Anxiety *M* = 1.99. MENAP = Middle East and North Africa Region plus Afghanistan/Pakistan, MB = migration background, QUAPT = *Questionnaire on Attitudes Toward Psychotherapeutic Treatment*, SCL = *Symptom Check List, SE =* standard error, Sum Sq = Sum of Squares, df = degrees of freedom, MSS = Mean sum of squares.

* *p* < .05

|  | QUAPT General Attitude | | | | | | | | |
| --- | --- | --- | --- | --- | --- | --- | --- | --- | --- |
| Migration background | Corrected estimated mean value (corrected *SE)* | | | | | *p*-value | | | |
|  | Analysis 1 | | | Analysis 2 | | Analysis 1 | | Analysis 2 | |
| No MB | 2.98 (0.05) | | | 3.07 (0.05) | |  | |  | |
| Turkish MB | 3.11 (0.06) | | | 3.06 (0.07) | | .261 | | 1.000 | |
| Eastern European MB | 3.06 (0.10) | | | 3.08 (0.11) | | .865 | | .999 | |
| MENAP MB | 2.94 (0.11) | | | 2.81 (0.12) | | .978 | | .153 | |
| Variable | | Sum Sq | *Df* | | MSS | | F | | *p*-value |
| Covariates analysis 1 :  Gender | | 2.96 | 1 | | 2.96 | | 7.50 | | .006* |
| Independent variable:  Migration background | | 1.36 | 3 | | 0.45 | | 1.15 | | .328 |
| Residuals | | 143.06 | 363 | | 0.39 | |  | |  |
| Covariates analysis 2:  Gender  SCL Somatization | | 2.80  1.67 | 1  1 | | 2.80  1.67 | | 7.94  4.73 | | .005*  .030* |
| SCL Depression  School education  Religious affiliation  Inpatient stays  Independent variable: | | 0.33  3.31  0.72  3.09 | 1  1  1  1 | | 0.33  3.31  0.71  3.09 | | 0.94  9.40  2.05  8.78 | | .334  .002*  .153  .003* |
| Migration background | | 1.51 | 3 | | 0.50 | | 1.43 | | .236 |
| Residuals | | 104.70 | 297 | | 0.35 | |  | |  |

**Table S4.** Results and descriptive statistics of the two analyses of covariance with the factor migration background and the QUAPT general attitude scale as dependent variable. *R*² = .11*, corrected *R*² = .09 (both for analysis 2). The corrections are based on the mean value of SCL Somatization *M*=2.41, SCL Depression *M* = 2.83, SCL Anxiety *M* = 1.99. MENAP = Middle East and North Africa Region plus Afghanistan/Pakistan, MB = migration background, QUAPT = *Questionnaire on Attitudes Toward Psychotherapeutic Treatment*, SCL = *Symptom Check List, SE =* standard error, Sum Sq = Sum of Squares, df = degrees of freedom, MSS = Mean sum of squares.

* *p* < .05

|  | Drug Attitude Inventory | | | | | | | | |
| --- | --- | --- | --- | --- | --- | --- | --- | --- | --- |
| Migration background | Corrected estimated mean value (corrected *SE)* | | | | | *p*-value | | | |
|  | Analysis 1 | | | Analysis 2 | | Analysis 1 | | Analysis 2 | |
| No MB | 1.52 (0.02) | | | 1.51 (0.02) | |  | |  | |
| Turkish MB | 1.63* (0.02) | | | 1.63* (0.02) | | .001* | | < .001* | |
| Eastern European MB | 1.63* (0.04) | | | 1.64* (0.04) | | .023* | | .006* | |
| MENAP MB | 1.56 (0.04) | | | 1.57 (0.04) | | .719 | | .488 | |
| Variable | | Sum Sq | *Df* | | MSS | | F | | *p*-value |
| Covariates analysis 1:  Gender | | 0.26 | 1 | | 0.26 | | 4.17 | | .042* |
| Independent variable:  Migration background | | 0.98 | 3 | | 0.33 | | 5.30 | | .001* |
| Residuals | | 22.25 | 362 | | 0.06 | |  | |  |
| Covariates analysis 2:  Gender  SCL Depression  Medication intake  Inpatient stays  independent variable: | | 0.26  0.34  2.71  0.01 | 1  1  1  1 | | 0.26  0.34  2.71  0.01 | | 4.81  6.25  50.03  0.13 | | .029*  .013*  < .001*  .717 |
| Migration background | | 1.02 | 3 | | 0.34 | | 6.28 | | < .001* |
| Residuals | | 18.52 | 342 | | 0.05 | |  | |  |

**Table S5.** Results and descriptive statistics of the two analysis of covariance with the factor migration background and the Drug Attitude Inventory (DAI) as dependent variable.*R*² = .19*, corrected *R*² = .17 (both for analysis 2). The corrections are based on the mean value of SCL Somatization *M*=2.41, SCL Depression *M* = 2.83, SCL Anxiety *M* = 1.99. The DAI value represents an arithmetic mean of a 2 point Likert scale (1 = True*,* 2 = False) with higher values indicating a more positive attitude. MENA = Middle East and North Africa Region, MG = migration background, DAI = *Drug Attitude* *Inventory*, SCL = *Symptom Check List, SE =* standard error, Sum Sq = Sum of Squares, df = degrees of freedom, MSS = Mean sum of squares.

* *p* < .05

|  | Turkish background (N=111) | | | | | | | Eastern European background (N=39) | | | | | | | MENAP background (N=37) | | | | | | |
| --- | --- | --- | --- | --- | --- | --- | --- | --- | --- | --- | --- | --- | --- | --- | --- | --- | --- | --- | --- | --- | --- |
| Dependent variable | **B (95%CI)** | **β** | **R^2^** | **∆R^2^** | **F for ∆R^2^** | **P for ∆R^2^** | **n** | **B (95%CI)** | **β** | **R^2^** | **∆R^2^** | **F for ∆R^2^** | **P for ∆R^2^** | **n** | **B (95%CI)** | **β** | **R^2^** | **∆R^2^** | **F for ∆R^2^** | **P for ∆R^2^** | **n** |
| QAPT judgment |  |  |  |  |  |  |  |  |  |  |  |  |  |  |  |  |  |  |  |  |  |
| Step 1: |  |  | .17* | .17 | 3.83 | .003* |  |  |  | .27 | .27 | 1.66 | .186 |  |  |  | .16 | .16 | 0.86 | .523 |  |
| SCL Somatization | 0.01 (-0.15-0.17) | .02 |  |  |  |  |  | -0.24 (-0.57-0.09) | -.37 |  |  |  |  |  | -0.24 (-0.65-0.17) | -.30 |  |  |  |  |  |
| SCL Anxiety | 0.11 (-0.04-0.27) | .17 |  |  |  |  |  | -0.03 (-0.44-0.38) | -.04 |  |  |  |  |  | -0.01 (-0.33-0.32) | -.01 |  |  |  |  |  |
| SCL Depression | -0.34* (-0.49- -0.14) | -.48 |  |  |  |  |  | -0.08 (-0.46-0.30) | -.10 |  |  |  |  |  | -0.06 (-0.34-0.47) | .08 |  |  |  |  |  |
| Religious affiliation | -0.05 (-0.52-0.42) | -.02 |  |  |  |  |  | 0.00 (-0.74-0.73) | .00 |  |  |  |  |  | -0.30 (-1.20-0.61) | -.14 |  |  |  |  |  |
| Medication intake | -0.05 (-0.49-0.38) |  |  |  |  |  |  | -0.77 (-2.52-0.98) | -.17 |  |  |  |  |  | -0.60 (-1.75-0.56) | -.21 |  |  |  |  |  |
| Step 2: |  |  | .20* | .03 | 1.91 | .154 |  |  |  | .50 | .23 | 4.61 | .022* |  |  |  | .19 | .03 | 0.42 | .662 |  |
| Host national identification | 0.00 (-0.12-0.11) | .00 |  |  |  |  |  | 0.17 (-0.13-0.47) | .27 |  |  |  |  |  | -.08 (-0.44-0.28) | -.13 |  |  |  |  |  |
| Co-national identification | -0.12 (-0.24-0.00) | -.20 |  |  |  |  |  | 0.25 (-0.08-0.59) | .37 |  |  |  |  |  | 0.12 (-0.16-0.40) | .22 |  |  |  |  |  |
|  |  |  |  |  |  |  | 100 |  |  |  |  |  |  | 26 |  |  |  |  |  |  | 29 |
| QAPT competence |  |  |  |  |  |  |  |  |  |  |  |  |  |  |  |  |  |  |  |  |  |
| Step 1: |  |  | .03 | .03 | 1.36 | .263 |  |  |  | .12 | .12 | 1.50 | .244 |  |  |  | .07 | .07 | 1.07 | .359 |  |
| School education | 0.17 (-0.04-0.37) | .17 |  |  |  |  |  | 0.48 (0.30-1.23) | .33 |  |  |  |  |  | -0.25 (-0.67-0.16) | -.23 |  |  |  |  |  |
| Inpatient stays | 0.00 (-0.14-0.15) | .00 |  |  |  |  |  | 0.05 (-0.47-0.21) | .05 |  |  |  |  |  | -0.13 (-0.42-0.16) | -.18 |  |  |  |  |  |
| Step 2: |  |  |  |  |  |  |  |  |  |  |  |  |  |  |  |  |  |  |  |  |  |
| Host national identification | 0.01 (-0.07-0.10) | .02 |  |  |  |  |  | -0.27 (-0.59-0.06) | -.51 |  |  |  |  |  | -0.01 (-0.22-0.20) | -.03 |  |  |  |  |  |
| Co-national identification | 0.007 (-0.02-0.15) | .16 |  |  |  |  |  | 0.15 (-0.18-0.48) | .26 |  |  |  |  |  | -0.01 (-0.18-0.20) | .02 |  |  |  |  |  |
|  |  |  |  |  |  |  | 90 |  |  |  |  |  |  | 26 |  |  |  |  |  |  | 30 |
| QAPT acceptance |  |  |  |  |  |  |  |  |  |  |  |  |  |  |  |  |  |  |  |  |  |
| Step 1: |  |  | .07 | .07 | 3.09 | 0.51 |  |  |  | .35* | .35 | 6.10 | .008* |  |  |  | .01 | .01 | 0.11 | .897 |  |
| School education | 0.22 (-0.00-0.43) | .20 |  |  |  |  |  | 0.77* (0.30-1.23) | .58 |  |  |  |  |  | -0.06 (-0.46-0.33) | -.06 |  |  |  |  |  |
| Inpatient stays | -0.10 (-0.25-0.05) | -.13 |  |  |  |  |  | -0.13(-0.47-0.21) | -.14 |  |  |  |  |  | -0.05 (-0.33-0.23) | -.07 |  |  |  |  |  |
| Step 2: |  |  | .12* | .05 | 2.56 | .083 |  |  |  | .39* | .04 | .76 | .480 |  |  |  | .04 | .03 | 0.42 | .663 |  |
| Host national identification | -0.05 (-0.14-0.04) | -.11 |  |  |  |  |  | -0.15 (-0.42-0.12) | -.31 |  |  |  |  |  | -0.05 (-0.25-0.15) | -.14 |  |  |  |  |  |
| Co-national identification | 0.09 (-0.00-0.18) | .20 |  |  |  |  |  | 0.07 (-0.20-0.35) | .14 |  |  |  |  |  | 0.08 (-0.10-0.26) | .22 |  |  |  |  |  |
|  |  |  |  |  |  |  | 90 |  |  |  |  |  |  | 26 |  |  |  |  |  |  | 31 |
| QAPT general attitude |  |  |  |  |  |  |  |  |  |  |  |  |  |  |  |  |  |  |  |  |  |
| Step 1: |  |  | .19* | .19 | 3.12 | 0.008* |  |  |  | .47* | .47 | 2.71 | .047* |  |  |  | .02 | .02 | 0.07 | .998 |  |
| Gender | -0.33* (-0.61- -0.05) | -.24 |  |  |  |  |  | 0.39 (-0.18-0.96) | .29 |  |  |  |  |  | 0.00 (-0.82-0.81) | .00 |  |  |  |  |  |
| SCL Somatization | 0.11 (-0.03-0.24) | .24 |  |  |  |  |  | -0.02 (-0.26-0.22) | -0.04 |  |  |  |  |  | -0.15 (-0.66-0.36) | -.16 |  |  |  |  |  |
| SCL Depression | -0.01(-0.16-0.14) | -.02 |  |  |  |  |  | -0.18 (-0.49-0.13) | -.31 |  |  |  |  |  | 0.05 (-0.47-0.57) | .05 |  |  |  |  |  |
| School education | 0.16 (-0.08-0.41) | .14 |  |  |  |  |  | 0.68* (0.17-1.20) | .51 |  |  |  |  |  | 0.00 (-0.86-0.86) | .00 |  |  |  |  |  |
| Religious affiliation | -0.13 (-0.52-0.26) | -.07 |  |  |  |  |  | 0.32 (-0.25-0.90) | .23 |  |  |  |  |  | 0.02 (-1.17-1.20) | .01 |  |  |  |  |  |
| Inpatient stays | -0.17* (-0.34- -0.00) | -.21 |  |  |  |  |  | -0.08 (-0.50-0.44) | -.08 |  |  |  |  |  | -0.07 (-0.68-0.54) | -.06 |  |  |  |  |  |
| Step 2: |  |  | .19* | .00 | 0.34 | .715 |  |  |  | .51 | .04 | 0.51 | .607 |  |  |  | .10 | .08 | 0.81 | .459 |  |
| Host national identification | 0.02 (-0.08-0.13)( | .06 |  |  |  |  |  | 0.07 (-0.25-0.39) | .14 |  |  |  |  |  | -0.09 (-0.57-0.39) | -.12 |  |  |  |  |  |
| Co-national identification | 0.03 (-0.07-0.14) | .07 |  |  |  |  |  | -0.15 (-0.46-0.17) | -.27 |  |  |  |  |  | -0.16 (-0.53-0.21) | -.23 |  |  |  |  |  |
|  |  |  |  |  |  |  | 89 |  |  |  |  |  |  | 25 |  |  |  |  |  |  | 27 |
| Drug Attitude Inventory |  |  |  |  |  |  |  |  |  |  |  |  |  |  |  |  |  |  |  |  |  |
| Step 1: |  |  | .09 | .09 | 2.37 | .058 |  |  |  | .28 | .28 | 2.24 | 0.96 |  |  |  | .19 | .19 | 1.57 | .212 |  |
| Gender | -0.06 (-0.17-0.04) | -.12 |  |  |  |  |  | 0.02 (-0.17-0.22) | .05 |  |  |  |  |  | 0.08 (-0.08-0.24) | .19 |  |  |  |  |  |
| SCL Depression | -0.02 (-0.06-0.02) | -.12 |  |  |  |  |  | -0.02 (-0.10-0.06) | -.09 |  |  |  |  |  | -0.08 (-0.17-0.01) | -.37 |  |  |  |  |  |
| Medication intake | 0.19* (0.05-0.33) | .28 |  |  |  |  |  | 0.66* (0.13-1.19) | .50 |  |  |  |  |  | 0.17 (-0.16-0.50) | .19 |  |  |  |  |  |
| Inpatient stays | -0.03 (-0.10-0.03) | -.10 |  |  |  |  |  | 0.04 (-0.11-0.19) | .10 |  |  |  |  |  | 0.02 (-0.09-0.14) | .08 |  |  |  |  |  |
| Step 2 |  |  | .09 | .00 | 0.04 | .966 |  |  |  | .34 | .06 | 0.87 | .432 |  |  |  | .20 | .02 | 0.14 | .871 |  |
| Host national identification | 0.00 (-0.04-0.03) | -.02 |  |  |  |  |  | -0.03 (-0.13-0.08) | -.16 |  |  |  |  |  | 0.01 (-0.08-0.11) | .06 |  |  |  |  |  |
| Co-national identification | 0.00 (-0.04-0.03) | -.02 |  |  |  |  |  | -0.03 (-0.14-0.09) | -.13 |  |  |  |  |  | 0.01 (-0.06-0.08) | .06 |  |  |  |  |  |
|  |  |  |  |  |  |  | 98 |  |  |  |  |  |  | 28 |  |  |  |  |  |  | 31 |

**Table S6.** Complete results of the hierarchical Regression predicting the QUAPT scales judgment, competence, acceptance and general attitude as well as the DAI scale within the samples with Turkish, East European and MENAP background. The acculturation scales are added in the second step. School education: 0 = low, 1= high, Gender: 0 = female, 1 = male, religious affiliation: 0 = yes, 1 = no. Higher scores on the scales of the QUAPT and DAI indicate a more positive attitude on that scale. For simplicity reasons the control variables are only presented in step 1. MENA = Middle East and North Africa Region, QUAPT = *Questionnaire on Attitudes Toward Psychotherapeutic Treatment*, DAI = *Drug Attitude* *Inventory*, SCL = *Symptom Check List.** *p* < .05
